# Chitosan nanoparticles as antigen vehicles to induce effective tumor specific T cell responses

**DOI:** 10.1371/journal.pone.0239369

**Published:** 2020-09-30

**Authors:** Frederik Walter, Elsa Winter, Sascha Rahn, Judith Heidland, Saskia Meier, Anna-Maria Struzek, Marcus Lettau, Lisa-Marie Philipp, Silje Beckinger, Lilli Otto, Julia Luisa Möller, Ole Helm, Daniela Wesch, Regina Scherließ, Susanne Sebens

**Affiliations:** 1 Institute for Experimental Cancer Research, Kiel University and University Medical Center Schleswig-Holstein (UKSH) Campus Kiel, Kiel, Germany; 2 Institute of Biochemistry, Kiel University, Kiel, Germany; 3 Department of Pharmaceutics and Biopharmaceutics, Kiel University, Kiel, Germany; 4 Institute of Immunology, Kiel University and UKSH Campus Kiel, Kiel, Germany; 5 Department of Hematology and Oncology, University Medical Center Schleswig-Holstein (UKSH) Campus Kiel, Kiel, Germany; University of Pécs Medical School, HUNGARY

## Abstract

Cancer vaccinations sensitize the immune system to recognize tumor-specific antigens *de novo* or boosting preexisting immune responses. Dendritic cells (DCs) are regarded as the most potent antigen presenting cells (APCs) for induction of (cancer) antigen-specific CD8+ T cell responses. Chitosan nanoparticles (CNPs) used as delivery vehicle have been shown to improve anti-tumor responses. This study aimed at exploring the potential of CNPs as antigen delivery system by assessing activation and expansion of antigen-specific CD8+ T cells by DCs and subsequent T cell-mediated lysis of pancreatic ductal adenocarcinoma (PDAC) cells. As model antigen the ovalbumin-derived peptide SIINFEKL was chosen. Using imaging cytometry, intracellular uptake of FITC-labelled CNPs of three different sizes and qualities (90/10, 90/20 and 90/50) was demonstrated in DCs and in pro- and anti-inflammatory macrophages to different extents. While larger particles (90/50) impaired survival of all APCs, small CNPs (90/10) were not toxic for DCs. Internalization of SIINFEKL-loaded but not empty 90/10-CNPs promoted a pro-inflammatory phenotype of DCs indicated by elevated expression of pro-inflammatory cytokines. Treatment of murine DC2.4 cells with SIINFEKL-loaded 90/10-CNPs led to a marked MHC-related presentation of SIINFEKL and enabled DC2.4 cells to potently activate SIINFEKL-specific CD8+ OT-1 T cells finally leading to effective lysis of the PDAC cell line Panc-OVA. Overall, our study supports the suitability of CNPs as antigen vehicle to induce potent anti-tumor immune responses by activation and expansion of tumor antigen-specific CD8+ T cells.

## Introduction

During cancer progression cancer cells develop various strategies by which they escape and impair the attack by the immune system [1]. Thus, the basic principles of current immune therapies are targeting of regulatory/immunosuppressive mechanisms and inducing/restoring immunity against the cancer [2–4]. Cancer vaccinations aim at sensitizing the patient`s immune system to recognize tumor-specific antigens *de novo* or boosting preexisting immune responses with the ultimate goal to induce long-term tumor-specific CD8+ T cell responses [2, 5, 6]. In this context, the therapeutic efficacy is highly dependent on a sufficient and proper presentation of cancer antigens on major histocompatibility complexes (MHC)-I and -II by antigen presenting cells (APCs) to elicit activation and effector function of tumor-reactive CD8+ and CD4+ T lymphocytes [6, 7]. Dendritic cells (DCs) are regarded as the most potent APCs for induction of (cancer) antigen-specific CD8+ T cell responses [8]. Many studies have already demonstrated that pulsing of DCs with MHC-I restricted tumor-derived peptides or whole tumor cell lysates leads to induction of CD8+ T cell-mediated anti-cancer responses *in vitro* and *in vivo* [7]. DCs can exhibit different phenotypes in dependence on the environmental conditions. Hence, in response to specific factors DCs mature and thereby become enabled to mediate T cell priming and activation. In this context, it has been shown that the adjuvant component of vaccines is a critical determinant in triggering DC maturation [9]. Different strategies have been explored in order to optimize antigen presentation by DCs, e.g. DC isolation combined with *ex vivo* antigen pulsing or *in vivo* vaccination [10].

Formulation of antigens into biocompatible delivery systems has been shown to significantly increase bioavailability of antigens as well as their uptake and processing by DCs leading to improved anti-cancer responses [11–16]. Chitosan is a polysaccharide (deacetylated chitin) derived primarily from crustaceans and exhibits adjuvant/ pro-inflammatory properties by which it is able to induce an innate immune response [17]. In the context of antigen-specific immune responses, chitosan shows adjuvant activity and as such is an interesting biopolymer to be used in a (tumor) vaccination setting [18, 19]. The prerequisites for its adjuvant activity are still not fully understood and results are inconclusive regarding the influence of molecular weight and degree of deacetylation (DDA) on its adjuvant activity. Since previous studies revealed an advantage of chitosan with 90% DDA over other DDAs, thus, this quality was also used for the present study [20].

Moreover, chitosan has already been described as a suitable delivery system for vaccination [12–16, 21–24] and chitosan nanoparticles (CNPs) have already been used to improve anti-tumor responses [25–27]. First approaches making use of CNPs as cellular delivery system e.g. for chemotherapeutic drugs have also been tested in preclinical models of pancreatic ductal adenocarcinoma (PDAC) with encouraging results [28, 29]. Chitosan nanoparticles can be transferred to immune competent cells utilizing various routes including the mucosal route. This is a very promising approach, especially if an intense cytotoxic immune response is needed as in the case of tumor vaccination, as the mucosal immune system fosters cellular immune response over humoral effects. Another advantage of utilizing the mucosal route is its non-invasive application (compared to injection). Indeed, CNPs can be delivered to the respiratory mucosa by oral inhalation or nasal administration if formulated to a drug product of the right characteristics [25]. In this study, CNPs have been stabilized in a dry powder mannitol matrix by spray drying to increase storage stability. By tuning the particle size of such a product, lung delivery (aerodynamic particle size below 5 μm) or nasal deposition (particle size > 10 μm) can be facilitated.

PDAC is the 4th most frequent cause of cancer related deaths in western countries and has still a poor overall 5-year-survival rate below 10%. Owing to the fact that reliable tests for early detection and disease specific symptoms are lacking, PDAC is commonly diagnosed in an advanced stage [30, 31]. Moreover, to date no effective therapeutic options are available for the treatment of patients with advanced PDAC stages, a fact that is owed to the profound resistance of the tumor to any available classical or targeted therapy [31–33]. One factor contributing to this broad therapy resistance is the pronounced tumor stroma that shows an exceptional intra- and inter-tumoral heterogeneity with respect to the abundance and distribution of various immune cell populations [33–35]. In contrast to other tumor entities like melanoma, PDAC is regarded as a poorly immunogenic tumor due to its low mutational burden [36–38]. Thus, a potent antigen delivery strategy leading to efficient antigen presentation by DCs and activation of specific CD8+ T cell clones represents a promising therapeutic strategy to induce a potent and long lasting PDAC directed immunity finally leading to the elimination or at least control of the tumor burden.

Making use of the ovalbumin-derived peptide SIINFEKL (OVA 257–264) as a model antigen, the aim of this study was to explore in a comprehensive *in vitro* approach the potential of CNPs of different sizes and qualities as antigen delivery system to induce proper activation of antigen-specific CD8+ T cells by DCs and subsequent T cell-mediated tumor cell lysis.

## Materials & methods

### Generation and characterization of CNPs

Chitosan nanoparticles were prepared by ionic gelation using different chitosan qualities (Chitoscience 90/10 (#23601), Chitoscience 90/20 (#23602) and Chitoscience 90/50 (#23603) all from Heppe Biomedical, Halle, Germany) with a DDA of 90% and different molecular weights (given as viscosity of a 2% solution in acetic acid being 10, 20 and 50 mPas). The quality is encrypted in the respective name X/Y where X stands for the DDA and Y for the respective viscosity. Chitosan has been labelled with FITC (#F7-250-1G, Sigma-Aldrich Chemie GmbH, Munich, Germany) to allow detection of the particles following the procedure as described [39]. Carmellose-sodium (CMC # C5678-500G, Sigma-Aldrich Chemie GmbH, Munich, Germany) was used as counter ionic polymer [40]. Chitosan was dissolved in 1% acetic acid obtaining a 0.1% w/w solution. CMC was dissolved in water, also obtaining a 0.1% w/w solution. Then, the CMC solution was added to the chitosan solution under constant stirring at room temperature (RT), which induced spontaneous self-assembly to nanoparticles. Afterwards, the dispersion was stored in the fridge (2–8 °C) for at least 3 hours to facilitate solidification of the nanoparticles before washing by centrifugation. The nanoparticles were resuspended again to a concentration of 0.1% w/w in 1% acetic acid before adding 2% mannitol (# 450001D, Roquette, France). The preparation was spray dried with the Mini Spray Dryer B-290, a lab-scale spray dryer, (Büchi, Flawil, Switzerland) using a two-fluid nozzle with a diameter of 1.5 mm. The spray gas flow was kept constant at 472 l/h and the volume flow was kept constant at 35 m³/h. The inlet temperature was set to 80 °C and the feed rate was adjusted to reach an outlet temperature lower than 40 °C (optimum: 35 °C). Particle size of the nanoparticle dispersion was determined by Dynamic Light Scattering (ZetaSizer, Malvern, UK). Used CNPs and their properties are listed in Table 1.

**Table 1:** CNP sizes after nanoparticle preparation and purification (z-Average is the mean particle size, polydispersity index (PDI) is a measure for width of distribution).

CNPs (with FITC-conjugation)	z-Average (in nm)	PDI
90/10	220	<0.17
90/20	384	0.22
90/50	706	0.39
90/10-OVA	201	0.14
90/20-OVA	321	0.15
90/10-SIINFEKL (without FITC-conjugation)	211	0.15

### Cell lines and cell culture

DC2.4 cells are immortalized murine DCs derived from C57BL/6 mice that have the ability to present antigens on MHC I comparable to human DCs [41]. DC2.4 cells are a kind gift from K.L. Rock, Dana-Farber Cancer Institute, Inc., Boston, Massachusetts. H441 is an immortalized cell line obtained from human papillary lung adenocarcinoma used as model for lung epithelium [42]. The cell line was kindly donated by Prof. Dr. Sabine Fuchs, Department of Trauma and Orthopedic Surgery, Experimental Trauma Surgery, University Medical Center Schleswig-Holstein, Kiel, Germany. Panc02 cells are murine malignant pancreatic ductal epithelial cells originating from C57BL/6 mice [43, 44] and Panc-OVA cells overexpressing SIINFEKL are derived from Panc02 cells. Both cell lines were kindly donated by Dr. Christian Bauer, Division of Gastroenterology, Endocrinology, Infectiology and Metabolism, University Hospital Giessen and Marburg, Campus Marburg, Philipps University Marburg, Germany. Cell lines were cultivated in RPMI 1640 medium (# R04-17500 supplemented with 10% FCS (# P30-1506, both PAN-Biotech, Aidenbach, Germany), 2 mM L-glutamine (#P04-80100, PAN-Biotech, Aidenbach, Germany) and 1% Pen/Strep (#15140–122, Gibco via Thermo Fisher Scientific, Schwerte, Germany). For DC2.4 cells, 1% minimum essential medium non-essential amino acids (# P08-32100, PAN-Biotech, Aidenbach, Germany) were added. For Panc-OVA cells medium was further supplemented with 0.5 mg/ml G418 sulfate (#A1720, Sigma-Aldrich Chemie, Munich, Germany). All cells were routinely cultured at 37 °C, 5% CO_2_ and 85% relative humidity. Absence of mycoplasma in cell cultures was regularly verified. Murine cell lines (DC2.4, Panc02 and Panc-OVA) were verified using DNA barcoding by PCR amplification of 5´coding region of cytochrome c oxidase I. Human H441 cells were verified by Short Tandem Repeat Analysis.

### Isolation of monocytes and generation of human antigen presenting cells

For *in vitro* generation of human APCs (M1- and M2-macrophages, DCs), monocytes were isolated from human thrombocyte-depleted lymphocyte retaining systems *via* density gradient centrifugation followed by counterflow centrifugation according to established protocols [45, 46]. Only blood from healthy donors was used and written informed consent was obtained from all donors. Approval was obtained by the ethics committee of the Medical Faculty at Kiel University (reference number: D490/17). Only monocytes with a purity of at least 90% were used for differentiation purposes. For differentiation into M1- or M2-macrophages, isolated monocytes were cultivated in RPMI 1640 medium supplemented with 1% FCS, 2 mM L-glutamine, 1% Pen/Strep and 2.4 ng/ml GM-CSF (240 U/ml, #572905) or 50 ng/ml M-CSF (100 U/ml, #574806, both Biolegend, Fell, Germany). Successful polarization of monocytes into M1- and M2-macrophages was assessed by flow cytometry and qPCR analysis (S1 Fig). After 7 days of differentiation culture in VueLife bags (#3300, CellGenix, Freiburg im Breisgau, Germany), macrophages were harvested and used for experiments. For generation of DCs [47], isolated monocytes were resuspended in RPMI 1640 medium supplemented with 10% FCS, 2 mM L-glutamine and 1% Pen/Strep and subsequently seeded into flat-bottom 12-well plates with 1 x 10^6^ cells/ml per well. After adherence, cells were stimulated with 250 U/ml IL-4 (#574004) and 800 U/ml GM-CSF (#572905, both Biolegend, Fell, Germany). Stimulation was repeated after 48 hours and differentiated DCs were used for experiments after 5 days of stimulation.

### Incubation of antigen presenting cells with CNPs

For uptake/internalization analyses of murine and human APCs, cells were either left untreated or treated with 100 μg/ml CNPs (90/10; 90/20; 90/50) for 24 hours. For this purpose, murine DC2.4 cells were seeded in flat-bottom 12-well plates at 8 x 10^4^ cells/ml per well 24 hours before stimulation. Human DCs were generated as described above. After a medium exchange, human DCs were differentially treated. M1- and M2-macrophages were also generated as described above and seeded at 2.5 x 10^5^ cells/ml per well in 12-well plates. After adherence, macrophages were differentially treated. Before analysis by flow cytometry or imaging cytometry, survival of cells was routinely checked using the EVOS XL Core Cell Imaging System (AMG, Bothell, USA). Surface markers used for characterization and identification of different APCs by flow cytometry and imaging cytometry are listed in S1 Table.

### Coculture of human DCs and H441 cells

Isolated human monocytes were seeded into 12-well plates at 1 x 10^6^ cells/ml per well and stimulated with IL-4 and GM-CSF as described above. After 5 days of differentiation culture, 1 x 10^5^ /ml H441 human lung epithelial cells were added per well to the (differentiated) human DCs. An epithelial phenotype of H441 cells was ensured after 24 hours in coculture and 100 μg/ml CNPs (90/10; 90/20; 90/50) were added. Internalization analyses were performed by imaging cytometry after 24 hours of CNP incubation. Surface markers used for characterization and identification of different cell populations are listed in S1 Table.

### Isolation of murine CD8+ T lymphocytes from OT-1 mice and coculture with antigen-pulsed DC2.4 cells

Animal experiments and care were carried out in accordance with European guidelines for care and use of laboratory animals and approved by the Ministry of Energy, Agriculture, Environment, Nature and Digitalization of Schleswig-Holstein (reference number 1115). After euthanasia by cervical dislocation, spleens from OT-1 mice were removed and mechanically crushed through a Falcon cell strainer (mesh size 100 μm, #352360, Falcon via Thermo Fisher Scientific, Schwerte, Germany). Afterwards, splenocytes were washed with ice-cold MACS buffer (PBS supplemented with 0.5% BSA, 2 mM EDTA, pH 7.4, sterile filtered and degassed) and cell suspension was filtered through a Falcon cell strainer (mesh size 30 μm, #352340, Falcon via Thermo Fisher Scientific, Schwerte, Germany). Then, splenocytes were centrifuged for 10 min at 300 x g and 4 °C, resuspended in erythrocyte lysis buffer (155 mM NH_4_Cl, 10 mM KHCO_3_, 0.1 mM EDTA in double-distilled water, sterile filtered) and incubated for 6 min at RT in the dark. Subsequently, splenocytes were washed with ice-cold MACS buffer, centrifuged for 10 min at 180 x g and 4 °C, resuspended in ice-cold MACS buffer and again filtered through a cell strainer (mesh size 30 μm). Afterwards, cell number was determined and cell suspension was adjusted to 10^8^ cells/ml. Hereafter, CD8+ OT-1 T lymphocytes were isolated from OT-1 splenocytes using the negative selection MojoSort Mouse CD8 T cell Isolation Kit (# 480011, Biolegend, Fell, Germany) according to the manufacturer’s instruction. After the isolation process, CD8+ OT-1 T cell purity was routinely analyzed via flow cytometric analysis. Only a CD8+ OT-1 T cell suspension with a purity > 90% were used for following experiments.

For coculture of CD8+ OT-1 T cells with DCs, DC2.4 cells were seeded one day before into 12-well cell culture plates as described before. DC2.4 cells were either left untreated or were stimulated with 100 μg/ml empty CNPs (90/10), 100 μg/ml SIINFEKL-loaded 90/10-CNPs or 1 μg/ml SIINFEKL (#vac-pova-100 InvivoGen Europe, Toulouse, France). After five hours, DC2.4 cells were detached, washed and then 7.5 x 10^4^ cells per well were seeded into flat-bottom 96-well plates for the coculture. Cell surface levels of SIINFEKL bound to H-2Kb complex in differentially treated DC2.4 cells were routinely analyzed by flow cytometry before starting the coculture with isolated CD8+ OT-1 T cells. Finally, 2.5 x 10^5^ CD8+ OT-1 T cells were added per well of a 96-well plate to the DC2.4 cells as well as 150 ng/ml recombinant murine IL-2 (562.5 U/ml, #575404, Biolegend, Fell, Germany). Restimulation of T cells with 150 ng/ml recombinant murine IL-2 (562.5 U/ml) was performed 48h after starting the coculture. 72 hours after coculture start, CD8+ OT-1 T cells were subjected to vital cell counting using the Trypan blue (#15250061, Gibco via Thermo Fisher Scientific, Schwerte, Germany) exclusion method and a Neubauer Chamber, flow cytometric analysis of T cell activation markers and tumor cell killing assay. Surface markers used for characterization and identification of different cell populations are listed in S1 Table.

### Killing assays with CD8+ OT-1 T lymphocytes

In order to analyze antigen-specific tumor cell lysis, Panc02 and Panc-OVA cells, respectively, were seeded at a density of 1 x 10^3^ cells per well in a 96-well plate. The next day, 2.0 x 10^5^ CD8+ OT-1 T lymphocytes, which has been shown in titrating experiments to be a reasonable cell number for this experimental approach, from different coculture settings with DC2.4 cells were added per well. After 24 hours, killing of pancreatic tumor cells was evaluated using the Lionheart FX Automated Microscope (BioTek, Bad Friedrichshall, Germany). For this purpose, CD8+ OT-1 T cells as well as detached Panc02 and Panc-OVA cells, respectively, were carefully aspirated and sterile, preheated PBS was added to the wells. For analysis of the cellular confluence, wells were scanned at 4-fold magnification and images were stitched with the Gen5 Data Analysis Software (BioTek, Bad Friedrichshall, Germany) to generate one picture of the whole well. Afterwards, a representative square plug with the size of 9,000 μm^2^ was selected *via* the implemented “Cellular Analysis” tool. Finally, quantification of cellular confluence was performed by using the Gen5 Data Analysis Software which discriminated the cellular area from cell-free area based on the obtained phase contrast signals and adjustment of specific parameters of the “Cellular Analysis” tool (see Table 2):

**Table 2:** Parameters of the “Cellular Analysis” tool used for determination of cellular confluence.

Parameter	Value
Threshold	Auto (checked) = 13
Background	light
Split touching objects	checked
Fill holes in the mask	unchecked
Min. object size	10 μm
Max. object size	10.000 μm
Include primary edge objects	checked
Analyze entire image	unchecked
	plug shape = square
	plug size = 3.000 μm x 3.000 μm
**Advanced detection options -**	
Background flattening	checked
Auto	unchecked
Rolling ball diameter	10 μm
Image smoothing strength	10 cycles
Evaluate background on	0% of lowest pixels
Primary mask	Use threshold mask

Cellular confluence in the representative area was calculated by the following formula:

$$\text{Cellular}\;\text{confluence}\;(\%)=\frac{{\text{Object}\;\text{sum}\;\text{area}\;(\mu \text{m}}^{2}\text{)}}{{\text{Plug}\;\text{size}\;(\mu \text{m}}^{2}\text{)}}\cdot 100$$

### Flow cytometry

Assessment of cell differentiation, lineage markers and T cell activation markers as well as cell surface SIINFEKL presentation via H-2Kb of OT-1 T cells and different APCs, respectively, was performed by immunofluorescence staining and subsequent flow cytometric analysis (markers used are listed in S1 Table). Prior to immunostaining, cells were incubated for 10 min at 4 °C in FcR Blocking Reagent (#130-059-901, Miltenyi Biotec, Bergisch Gladbach, Germany) diluted in ice-cold MACS buffer according to the manufacturer’s instructions. Afterwards, 2–4 x 10^5^ cells/well and staining were transferred to a 96-well V-bottom plate for immunofluorescence staining. For human DC phenotyping, anti-HLA-DR-FITC (clone: L243, #307603), anti-CD80-APC (clone: 2D10, #305220), anti-CD86-AlexaFluor488 (clone: IT2.2, #305413) (all Biolegend, Fell, Germany) and anti-PD-L1-PECy7 (clone: MIH1, #550017) (BD Biosciences, Heidelberg, Germany) were used. For characterization of human M1- and M2-macrophages, anti-CD14-PE (clone: M5E2, #301806), anti-CD16-FITC (clone: 3G8, #302006), anti-CD68-APC (clone: Y1/82A, #333810), anti-HLA-DR-FITC (clone: L243, #307603) (all Biolegend, Fell, Germany) and anti-CD163-PE (clone: REA812, #130-112-286 from Miltenyi Biotec, Bergisch Gladbach, Germany) were used. Anti-CD25-APC (clone: BC96, #302610), anti-CD44-FITC (clone: IM7, #103022), anti-CD69-PE (clone: H1.2F3, #104508) and anti-CD8a-PE (clone: 53–6.7, #100722) (all Biolegend, Fell, Germany) were used for phenotyping of murine CD8+ OT-1 T cells. To assess H-2Kb associated SIINFEKL presentation anti-SIINFEKL-H-2kb-PE (clone: 25-D1.16, #141604, Biolegend, Fell, Germany) was used. All antibodies were diluted in ice-cold MACS buffer according to the manufacturer’s instructions and incubated for 30 min at 4 °C in the dark. Specificity was verified by additional staining with respective isotype control antibodies. Data acquisition was performed with a FACScalibur making use of the CellQuest Pro software (both Becton Dickinson, San Jose, US). Final data evaluation was performed with FlowJo V10.1 software (FlowJo LCC, Oregon, US).

### Imaging cytometry

Imaging cytometry was used to determine internalization of CNPs. For membrane staining of human DCs anti-CD11c-APC (clone: S-HCL-3, #371506) was used while H441 cells were stained with anti-E-Cadherin-AlexaFluor647 (clone: 67A4, #324112) (both purchased from Biolegend, Fell, Germany). Murine DC2.4 cells were stained with anti-CD11c-PE (clone: HL3, #561044, BD Bioscience, Heidelberg, Germany). After staining and washing, cells were fixed in 50 μl 1% (v/v) PFA/MACS buffer solution. Imaging cytometry analyses were performed with the Amnis ImageStream^®X^ Mk II Imaging Flow Cytometer (Merck Millipore, Darmstadt, Germany).

The IDEAS^®^ Image analysis software was used for defining the cell boundary based on membrane staining (or brightfield images) so that cells with clear green intracellular fluorescence signal could be identified as cells with internalized FITC-conjugated CNPs whereas cells without intracellular fluorescence signal were identified as cells without CNP-internalization. The proportion of intracellularly stained cells was determined by calculating the ratio of cells with internalized CNPs to the total cell count.

### Confocal laser scanning microscopy (CLSM)

Confocal laser scanning microscopy (CLSM) analysis was used as another method to confirm internalization of CNPs in DCs. Isolated monocytes were seeded on cover slips (1 x 10^6^ cells/ ml) and differentiated to human DCs (as described before). After incubation with 100 μl/ml FITC-conjugated 90/10-CNPs for 24 hours, cells were washed with PBS and fixed with 400 μl of 4.5% PFA for 15 minutes at RT. After the next washing steps, 500 μl of 4% BSA/PBS were added and incubated for 30 minutes to block non-specific binding of immunoglobulins. Afterwards, anti-CD11c-APC (clone: S-HCL-3, #371506, Biolegend, Fell, Germany) for membrane staining of DCs and the nuclear staining dye Hoechst #33258 (#861405, Sigma-Aldrich, Munich, Germany) were diluted (anti-CD11c-APC 1:50, Hoechst 1:500) in 100 μl 1% BSA/PBS and cells were incubated for 60 minutes at room temperature protected from light. After washing, cover slips were mounted with FluorSave^™^ reagent (#345789, Merck Millipore, Darmstadt, Germany) and inverted onto glass slides. Overview images at lower magnifications (10X, 20X, 40X) were taken using the Lionheart FX Automated Microscope (BioTek, Bad Friedrichshall, Germany). Images at higher magnification (60X) were taken using CLSM (Carl Zeiss Microscopy GmbH, Jena, Germany).

### Caspase-3/7 activity assay

Caspase-3/7 activity in primary human DCs and macrophages was determined making use of the Caspase-3/7 Glo assay (#G8092, Promega, Mannheim, Germany) according to the manufacturer’s instructions. An identical number of cells for each cell type and experiment was seeded into the examined wells. All samples were analyzed in duplicates.

### Determination of cell confluence and PI stained cell area

For determining cell confluence and Propidium iodide (PI) stained cell area, human DCs that had been stimulated with 100 μg/ml CNPs (90/50; 90/20 and 90/10) for 24 hours, were stained with 20 μg/ml PI (#421301, Biolegend, Fell, Germany) diluted in fresh cell medium at RT protected from light. Imaging with SYNENTECs NYONE^®^ Scientific SC4 Cell Imager was performed after 24 hours. The resulting images were subsequently analyzed with the corresponding YT-software^®^.

### RNA isolation and RT-qPCR

Total RNA was isolated using the total RNA kit peqGOLD (#12-6834-02, PeqLab, Erlangen, Germany) and subjected to reverse transcription using Oligo dT Primer (# SO-132), Ribolock RNA Inhibitor (#EO-0382), dNTP-Mix (#RO-193) and Revert Aid M-MLV Reverse Transcriptase (#EP-0442) (all from Fermentas *via* Thermo Fisher Scientific, Darmstadt, Germany) according to manufacturer’s instructions. Quantitative Real-Time PCR analysis was performed as duplicate analysis on a LightCycler 480 (Roche Diagnostics, Mannheim, Germany) including melting curve analysis as quality control. Primers, primer sequences and annealing temperatures are listed in Table 3.

**Table 3:** Gene name as well as sequences, annealing temperatures and manufacturers of human and murine primers.

**Human primers**			
Gene	Primer sequence	Annealing°C	Manufacturer
GAPDH	Fw-TCCATGACAACTTTGGTATCGTGG	58	Eurofins, Hamburg, Germany
	Rv-GACGCCTGCTTCACCACCTTCT		
IL1-β	Fw-AGTGCTCCTTCCAGGACCTGGA	58	Eurofins, Hamburg, Germany
	Rv-CACTCTCCAGCTGTAGAGTGG		
IL-6	Fw-ATGCAATAACCACCCCTGAC	58	Realtime Primers, Elkins Park, US
	Rv-GAGGTGCCCATGCTACATTT		
IL-8	Fw- GTGTGAAGGTGCAGTTTTGCC	55	Eurofins, Hamburg, Germany
	Rv- AACTTCTCCACAACCCTCTGC		
IL-10	Fw-AAGCCTGACCACGCTTTCTA	58	Realtime Primers, Elkins Park, US
	Rv-ATGAAGTGGTTGGGGAATGA		
TNF-α	Fw-TCCTTCAGACACCCTCAACC	58	Eurofins, Hamburg, Germany
	Rv-AGGCCCCAGTTTGAATTCTT		
TGF-β1	Fw-CGTGGAGCTGTACCAGAAATA	58	Eurofins, Hamburg, Germany
	Rv-TCCGGTGACATCAAAAGATAA		
**Murine primers**			
Gene	Primer sequence	Annealing°C	Manufacturer
GAPDH	Fw-TCCATGACAACTTTGGTATCGTGG	58	Eurofins, Hamburg, Germany
	Rv-GACGCCTGCTTCACCACCTTCT		
mIL1-β	Fw-ATCCTCTGTGACTCATGGGAT	55	Biometra, Göttingen, Germany
	Rv-GATCCACACTCTCCAGCTGCA		
mIL-6	Fw-TAGTCCTTCCTACCCCAATTTCC	58	Eurofins, Hamburg, Germany
	Rv-TTGGTCCTTAGCCACTCCTTC		
mIL-10	Fw-AGTGGAGCAGGTGAAGAGTG	58	Realtime Primers, Elkins Park, US
	Rv-TTCGGAGAGAGGTACAAACG		
mTNF-α	Fw-CCCACTCTGACCCCTTTACT	58	Eurofins, Hamburg, Germany
	Rv-TTTGAGTCCTTGATGGTGGT		
mTGF-β1	Fw-GCTGAACCAAGGAGACGGAA	58	Eurofins, Hamburg, Germany
	Rv-AGAAGTTGGCATGGTAGCCC		

### Statistics

Statistical analyses were performed using SigmaPlot v12.5 provided by Systat. First, data were tested for normality and equal variance by Shapiro-Wilk and Equal Variance test, respectively. For comparison of two groups comprising parametric distributed datasets, t-test was applied. Two groups of datasets which failed normality or equal variance test were analyzed with Mann-Whitney Rank Sum test. Parametric data of multiple groups were checked with one-way analysis of variance (one-way ANOVA) for statistical significance. Non-parametrical datasets of multiple groups were analyzed with Kruskal-Wallis one-way ANOVA on ranks test. Statistically significant differences between the groups were assumed at p-values < 0.05 according to Student-Newman-Keuls method (parametric data) and Dunn’s method (non-parametric data), respectively. Statistically significant differences with p-values < 0.05 were marked with one asterisk *.

## Results

### Uptake of CNPs by different APC populations

**Fig 1:**
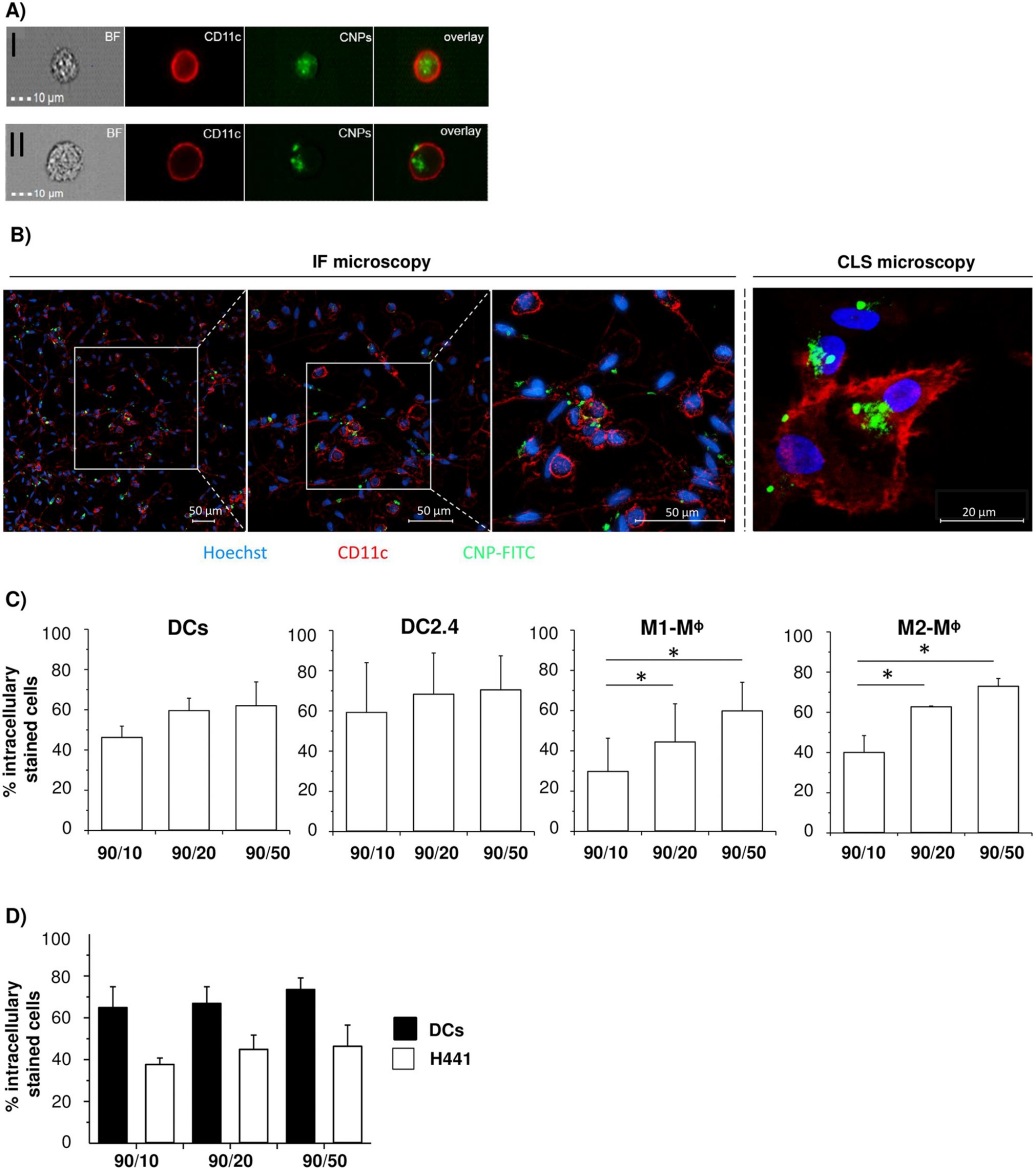
Uptake of CNPs by human and murine APCs. Different APC populations were incubated with 100 μg/ml CNPs of different size (90/10, 90/20 and 90/50) for 24 hours. **A**) Representative images of primary human dendritic cells (DCs) which were incubated with 90/10-CNPs and analyzed by Imaging cytometry; **I** shows DCs with internalized CNPs, **II** shows DCs with intra- and extracellular CNPs; BF = brightfield; red = CD11c-APC membrane staining of DCs; green = FITC-conjugated CNPs; overlay. **B)** Representative images of immunofluorescence (IF) and confocal laser scanning (CLS) microscopy of DCs incubated with 90/10-CNPs; blue = Hoechst nuclear counterstaining; red = CD11c-APC membrane staining; green = FITC-conjugated CNPs. **C)** Image cytometry analysis of CNP uptake by primary human DCs, murine DC line DC2.4 and human M1- or M2-macrophages (Mᶲ). **D)** Imaging cytometry analysis of CNP uptake by cocultured human DCs and human H441 lung epithelial cells after 24 hour incubation with CNPs. Data are expressed as % intracellularly stained cells and as mean + SEM of three independent experiments. *p < 0.05.

First, it was investigated whether CNPs are taken up by different APC populations and whether particle size is a critical determinant for uptake efficiency. For this purpose, human primary DCs, M1- and M2-macrophages as well as the murine DC line DC2.4 were incubated with 100 μg/ml CNPs for 24 hours. Chitosan qualities exhibiting varying molecular weights (90/10, 90/20 and 90/50) were utilized for CNPs generation, thereby resulting in different particle sizes. To set up analysis of CNP internalization by imaging cytometry, the cell membrane of human DCs was marked by immunofluorescence CD11c-APC cell surface staining, thereby allowing clear discrimination of intracellular uptake of FITC-labelled CNPs (Fig 1A, **upper row**) from CNPs only bound to the (cell) surface of human DCs (Fig 1A, **bottom row**). Intracellular 90/10-CNP uptake in human DCs was further analyzed by IF- and CLS microscopy confirming imaging cytometry as a suitable method to properly determine CNP uptake (Fig 1B). Thus, CNP uptake was analyzed in different APC populations by imaging cytometry revealing that CNPs of the three different sizes were taken up by all APC populations albeit to a variable extent (30–70%) (Fig 1C). Overall, the highest proportion of cells with internalized FITC-conjugated CNPs (60–70%) could be observed after incubation with the largest CNPs (90/50) in every cell population/line. Incubation with the smallest CNPs (90/10) led to a higher percentage of DCs (murine and human) showing CNP-internalization compared to both macrophage populations. Considering an airway application of CNP-based vaccine, it was next investigated whether CNPs are still efficiently taken up by DCs if embedded in a lung epithelial microenvironment. Interestingly, when human DCs were directly cocultured with human H441 epithelial cells representing the lung epithelial barrier, CNP uptake was observed in both populations. However, uptake was clearly higher in DCs (65–74% intracellularly stained cells) compared to H441 cells (38–46% intracellularly stained cells) (Fig 1D). Overall, these data indicate that CNPs of different sizes are efficiently taken up by APCs and best by DCs.

### Uptake of large CNPs is toxic for APCs

**Fig 2:**
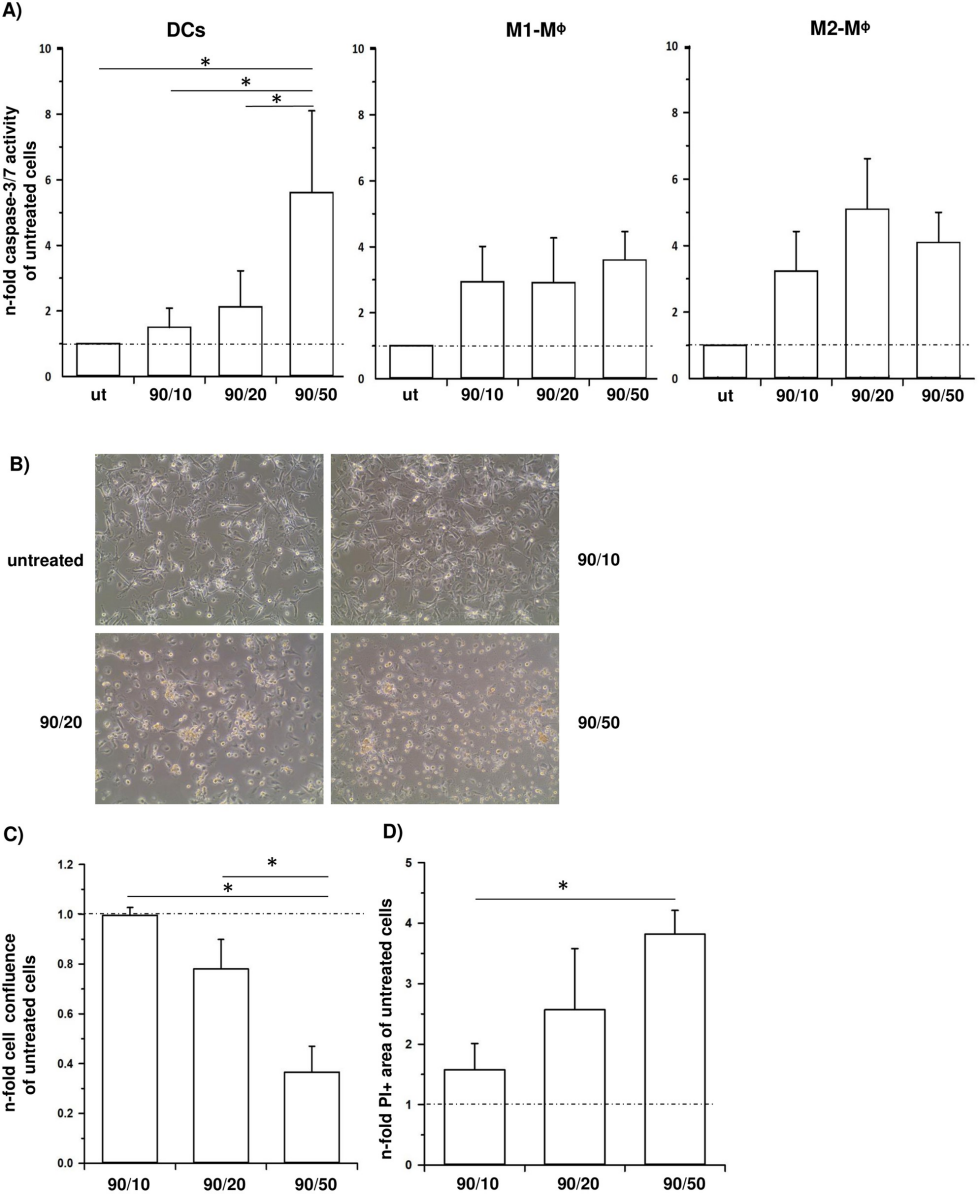
Uptake of large CNPs is toxic for APCs. Primary human DCs, M1- and M2-macrophages (Mᶲ) were either left untreated (ut) or incubated with 100 μg/ml CNPs of different size (90/10, 90/20 and 90/50) for 24 hours. **A**) Graphs present relative caspase-3/7 activity. Data are presented as n-fold caspase-3/7 activity of untreated cells and as median with 75th and 25th percentile of four independent experiments (DCs) or as mean + SEM of three independent experiments (M1- and M2-Mᶲ). **B**) Representative phase contrast images of DCs either left untreated or incubated for 24 hours with the indicated CNPs. Images were acquired at 280-fold magnification. **C-D**) DCs were stained with propidium iodide (PI) after indicated treatment and (**C**) cell confluence as well as (**D**) PI+ area were determined. Data are expressed as n-fold of untreated cells and mean + SEM of three independent experiments. *p < 0.05.

Next, it was investigated whether CNP uptake impacts survival of different APC populations. Therefore, human DCs, M1- and M2- macrophages were either left untreated or incubated with 100 μg/ml CNPs of different size (90/10, 90/20 and 90/50) for 24 hours and then analyzed for induction of cell death. First, caspase-3/7 activity was determined as an indicator for apoptosis induction. Compared to untreated cells a considerable (5-fold) increase in caspase-3/7 activity was observed in DCs only after incubation with 90/50-CNPs, while treatment with 90/20-CNPs caused a 2-fold increase and 90/10-CNPs even only an 1.5-fold increase (Fig 2A). In contrast, both macrophage populations showed a minimum 3-fold increased caspase-3/7 activity after incubation with CNPs of either size in comparison to untreated macrophages (Fig 2A). Since DCs are in the focus of our study, CNP-mediated induction of cell death was further examined in these cells. In line with the low caspase-3/7 activity, cell confluence of DCs treated with 90/10-CNPs was comparable to that of untreated cells (Fig 2B). However, a clearly decreased confluence was observed in wells with DCs that were treated with 90/20-CNPs, an effect which was even stronger after stimulation with 90/50-CNPs in comparison to the untreated control. Moreover, the remaining attached DCs in these wells did not display the typical dendritic cell morphology anymore that can be observed for untreated or 90/10-CNP treated cells (Fig 2B). Accordingly, detailed analyses of the differentially treated DCs by propidium iodide (PI) staining further confirmed these data and revealed significantly decreased cell confluence (Fig 2C) as well as a significant increase of the PI+ cell area with increasing CNPs size (Fig 2D). In summary, these data suggest that incubation with CNPs of increasing size impairs survival of APCs, particularly those of DCs. Since treatment with small CNPs (90/10) was best tolerated by DCs not impairing their cell viability, 90/10-CNPs were chosen for further experiments.

### Uptake of CNPs promotes a pro-inflammatory phenotype of DCs

**Fig 3:**
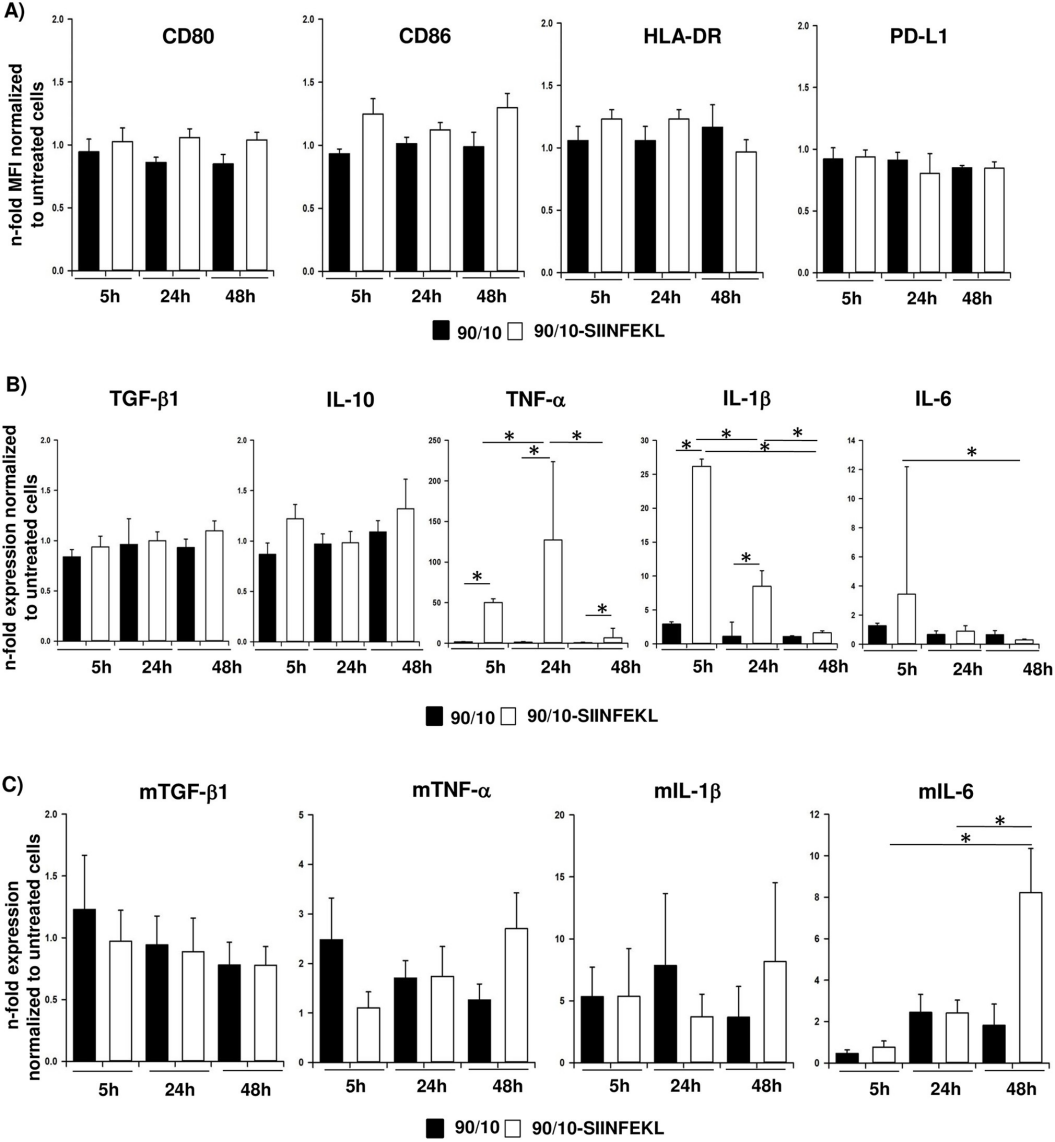
Uptake of CNPs promotes a pro-inflammatory phenotype of DCs. Human and murine DCs (DC2.4) were either left untreated or incubated with 100 μg/ml empty (90/10) or SIINFEKL-loaded 90/10-CNPs for 5, 24 and 48 hours. **A)** Flow cytometric analysis of CD80, CD86, HLA-DR and PD-L1 cell surface levels in human DCs. Median fluorescence intensity (MFI) ratio was calculated by normalizing MFI of each surface marker to the MFI of its respective isotype control. Then, MFI ratio of CNP treated cells was normalized to untreated cells and expressed as n-fold MFI of untreated cells. **B+C)** Relative mRNA levels of TGF-β1, IL-10, IL-1β, IL-6 and TNF-α in **B)** primary human DCs and **C)** murine DC2.4 cells was determined by RT-qPCR. Expression levels were normalized to expression of the housekeeping gene TBP/GAPDH and normalized to values determined for untreated cells. Data are presented as mean + SEM or median with 75^th^ and 25^th^ percentile of three independent experiments. *p < 0.05.

Having shown that 90/10-CNPs are taken up by ~ 50% of the population and least impair the survival of DCs, it was next investigated whether CNPs–empty or loaded with the model antigen SIINFEKL (OVA 257–264)–alter the phenotype of these cells. Thus, primary human DCs and the murine DC line DC2.4 were either left untreated or treated with 100 μg/ml empty (90/10) or SIINFEKL-loaded 90/10-CNPs (90/10-SIINFEKL) for 5, 24 and 48 hours, respectively. Flow cytometric analysis of the cell surface levels of costimulatory molecules CD80 and CD86 as well as HLA-DR and the coinhibitory protein PD-L1 revealed no considerable alterations in treated human DCs in comparison to untreated cells (Fig 3A, S2 Fig). Moreover, no clear effects were observed regarding the expression of anti-inflammatory cytokines such as TGF-β1 and IL-10 (Fig 3B). In contrast, the expression of the pro-inflammatory mediators IL-1β, IL-6 and TNF-α was strongly elevated after incubation with SIINFEKL-loaded 90/10-CNPs for 5 to 48 hours compared to incubation with empty CNPs (Fig 3B). Similar but less pronounced effects were observed in murine DC2.4 cells after incubation with peptide-loaded CNPs. Here, the expression levels of pro-inflammatory mediators IL-6, TNF-α and IL-1β were notably elevated and peaked 48 h after SIINFEKL-loaded 90/10-CNP addition and simultaneously reached markedly higher levels than after treatment with empty CNPs indicating an antigen-specific response (Fig 3C). As observed in human DCs, TGF-β1 expression was almost not affected (Fig 3C). Overall, these findings suggest that in human DCs particularly the SIINFEKL peptide but not the CNPs (and thus chitosan) themselves promote the acquisition of a pro-inflammatory phenotype while in the murine cell line DC2.4 both components foster the expression of pro-inflammatory cytokines as expected for an antigen-specific immune response.

### Uptake of SIINFEKL-loaded CNPs by DCs effectively stimulates antigen-specific activation and expansion of CD8+ OT-1 T lymphocytes

**Fig 4:**
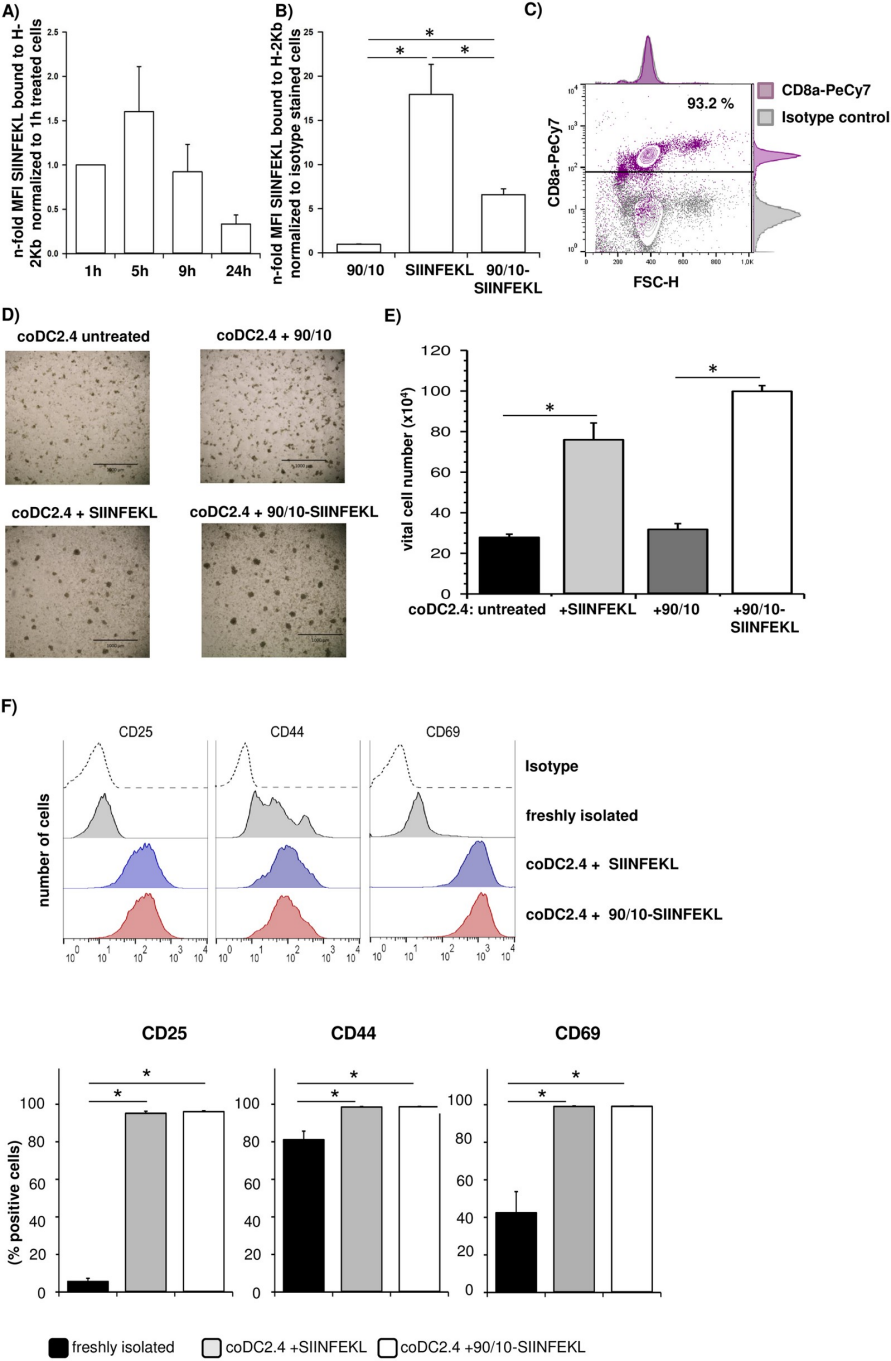
Uptake of SIINFEKL loaded CNPs by DCs efficiently stimulates CD8+ OT-1 T cell activation and expansion. **A)** DC2.4 cells were either left untreated or treated with 100 μg/ml SIINFEKL-loaded 90/10-CNPs for 1, 5, 9 and 24 hours or **B)** DC2.4 cells were treated with 100 μg/ml empty (90/10) or SIINFEKL-loaded 90/10-CNPs or 1 μg/ml SIINFEKL for 5 hours. SIINFEKL bound to H-2Kb molecules was determined by flow cytometric analysis. Median fluorescence intensity (MFI) ratio was calculated by normalizing MFI of staining with anti-SIINFEKL-H-2Kb antibody to MFI detected in staining with its respective isotype control (n-fold MFI). In **A)** MFI ratio of treated cells was also normalized to MFI of 1h treated cells and expressed as n-fold MFI of untreated cells. **C)** CD8+ OT-1 T lymphocytes were isolated from spleens of OT-1 mice. Purity of CD8+ OT-1 T cells was determined after negative MACS selection by CD8 staining and flow cytometric analysis. One representative dot blot is shown. **D-F)** DC2.4 cells were either left untreated or were treated with 100 μg/ml empty (90/10) or 100 μg/ml SIINFEKL-loaded 90/10-CNPs or 1 μg/ml SIINFEKL for 5 hours. Afterwards, differentially treated DC2.4 cells were cocultured with CD8+ OT-1 T lymphocytes for 72 hours. **D)** Representative phase contrast images of CD8+ OT-1 T cells cocultured with DC2.4 cells that had been either left untreated or subjected to the indicated treatment. Scale bar = 1000 μm. **E)** Numbers of vital CD8+ OT-1 T lymphocytes were determined after the indicated coculture setting. Data are presented as mean + SEM of three independent experiments. **F)** Flow cytometric analysis of T cell activation by staining of cell surface CD25, CD44 and CD69 of CD8+ OT-1 T cells directly after isolation and after coculture with DC.24 cells that had been pretreated with SIINFEKL or 90/10-SIINFEKL CNPs. Representative histograms from one out of 3 independent experiments are shown and bar charts present the % of CD8+ OT-1 T cells positive for cell surface expression of CD25, CD44 and CD69, respectively. Data are presented as mean +SEM of three independent experiments. *p < 0.05.

In order to induce a CD8+ T cell-mediated tumor directed immune response by CNP vaccination, loaded peptides have to be properly presented by MHC-I complexes on DCs leading to activation and expansion of antigen-specific CD8+ T cells. Incubation of DC2.4 cells with 100 μg/ml SIINFEKL-loaded 90/10-CNPs for different time-points revealed that the highest level of SIINFEKL bound to H-2Kb molecules was detectable after 5 hours (Fig 4A). Thus, all further experiments were analyzed after incubation with SIINFEKL-loaded CNPs for 5 hours. Verifying antibody specificity, SIINFEKL bound to H-2Kb complex was not detectable at the cell surface of DC2.4 cells after incubation with empty 90/10-CNPs for 5 hours (MFI Ratio = 1). In contrast, SIINFEKL bound to H-2Kb complex was highly present at the surface of DC2.4 cells incubated with the unpacked (soluble) SIINFEKL peptide (MFI Ratio = 17.5) or SIINFEKL-loaded 90/10-CNPs (MFI Ratio = 6) (Fig 4B).

In order to examine whether DC2.4 cells presenting SIINFEKL peptide *via* H-2Kb complex have the capability to stimulate CD8+ T cell activation, DC2.4 cells, either left untreated or incubated with unpacked SIINFEKL peptide, empty 90/10-CNPs or SIINFEKL-loaded 90/10-CNPs, were cocultured with CD8+ OT-1 T cells harboring a transgenic T cell receptor specific for H-2Kb-bound SIINFEKL. For this purpose, CD8+ OT-1 T cells were isolated from spleens of OT-1 mice by negative MACS selection providing untouched CD8+ OT-1 T cell populations of high purity (> 90%) (Fig 4C). Then, control and pre-treated DC2.4 cells were detached, washed to remove CNPs and SIINFEKL peptide that have not been taken up by DC2.4 cells and seeded for coculture. As soon as DC2.4 cells had attached to the culture dish surface, isolated CD8+ OT-1 T cells were added for co-culture and stimulated with mIL-2 which is essential for survival of potentially activated T cells. Light microscopic analyses after 72 hours of coculture showed that clusters of CD8+ OT-1 T cells had formed around DC2.4 cells in all four coculture settings. However, these clusters were much larger in number and size in coculture settings of CD8+ OT-1 T cells with DC2.4 cells that had been pretreated with unpacked SIINFEKL peptide or SIINFEKL-loaded 90/10-CNPs than in coculture settings with DC2.4 cells that had been untreated or pre-incubated only with empty 90/10-CNPs (Fig 4D). In line with these findings, vital cell numbers of CD8+ OT-1 T cells were significantly higher after coculture with DC2.4 cells pretreated with unpacked SIINFEKL or SIINFEKL-loaded 90/10-CNPs than after coculture with previously untreated or empty 90/10-CNP treated DC2.4 cells (Fig 4E). Moreover, flow cytometric analyses revealed a markedly increased proportion of CD8+ OT-1 T cells being positive for T cell activation markers CD25, CD44 and CD69 after coculture with DC2.4 cells that had been pretreated with unpacked SIINFEKL or SIINFEKL-loaded 90/10-CNP in comparison to freshly isolated CD8+ OT-1 T cells (Fig 4F). In summary, these data demonstrate that treatment of DC2.4 cells with SIINFEKL-loaded 90/10-CNPs led to a similarly potent activation and expansion of SIINFEKL-specific CD8+ OT-1 T cells as treatment with unpacked SIINFEKL peptide albeit expression of H-2Kb-bound SIINFEKL was higher after treatment with the unpacked SIINFEKL peptide.

### CD8+ OT-1 T cells activated by SIINFEKL presenting DCs after uptake of SIINFEKL-loaded CNPs efficiently kill SIINFEKL expressing tumor cells

**Fig 5:**
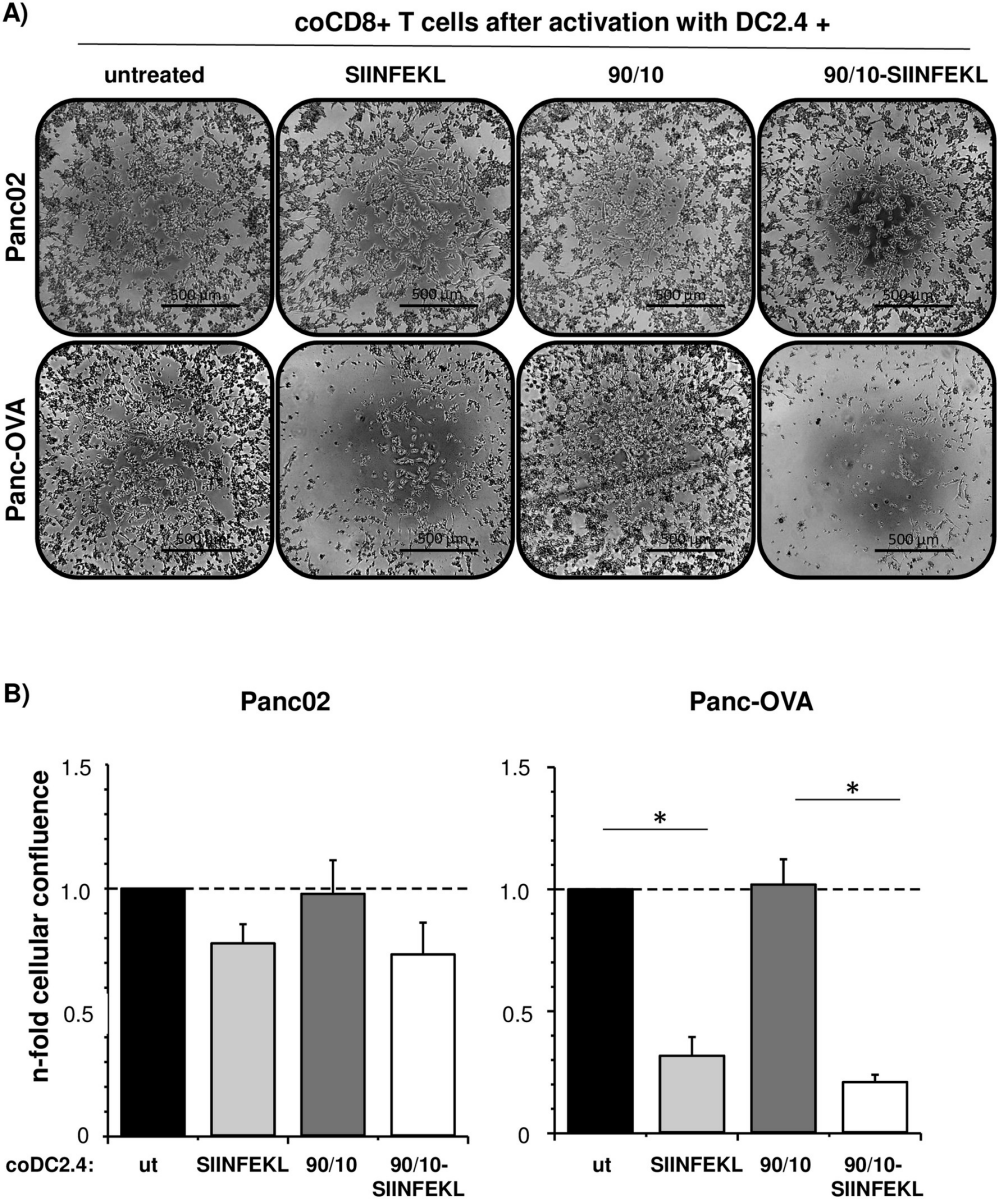
CD8+ OT-1 T cells activated by SIINFEKL presenting DCs after uptake of SIINFEKL-loaded CNPs efficiently kill SIINFEKL expressing tumor cells. Panc02 and Panc-OVA cells were cocultured with CD8+ OT-1 T lymphocytes derived from previous coculture with DC2.4 cells that had been either left untreated or treated with 100 μg/ml empty (90/10) or SIINFEKL-loaded 90/10-CNPs or 1 μg/ml SIINFEKL peptide. After 24 hours, CD8+ OT-1 T cells were removed from the wells and **A)** phase contrast images were taken (Scale bar = 500 μm) and **B)** cellular confluence of tumor cells was determined. Relative confluence of Panc02 and Panc-OVA cells after either indicated treatment is presented as n-fold of cellular confluence determined in cultures of respective Panc02 and Panc-OVA cells that were cultured with CD8+ OT-1 T lymphocytes from previous coculture with untreated DC2.4 cells. Data are presented as mean +SEM of three independent experiments. *p < 0.05.

Having shown that treatment of DC2.4 cells with SIINFEKL-loaded 90/10-CNPs led to an activation and expansion of CD8+ OT-1 T cells, it was next investigated whether these activated CD8+ T cells are able to kill tumor cells in an antigen-specific manner. For this purpose, the murine PDAC cell line Panc02 and its SIINFEKL expressing derivate Panc-OVA were cocultured with CD8+ OT-1 T cells derived from cocultures with differentially pretreated DC2.4 cells for 24 hours to analyze tumor cell killing. As shown in Fig 5A and 5B, confluence of both Panc02 and Panc-OVA cells was not affected by CD8+ OT-1 T cells from coculture with untreated or 90/10-CNP stimulated DC2.4 cells. In contrast, a reduced confluence of Panc02 and Panc-OVA cells was observed in the presence of CD8+ OT-1 T cells from coculture with DC2.4 cells pretreated with SIINFEKL peptide or SIINFEKL-loaded 90/10-CNPs. Notably, this reduction in cellular confluence was much more pronounced in Panc-OVA cells incubated with CD8+ OT-1 T cells from coculture with SIINFEKL (0.32-fold) or SIINFEKL-loaded 90/10-CNP treated DC2.4 cells (0.21-fold) than in Panc02 cells under similar conditions (SIINFEKL: 0.78-fold; 90/10-SIINFEKL: 0.73-fold). Overall, these data underscore that activation of CD8+ OT-1 T cells by DCs treated with SIINFEKL-loaded 90/10-CNPs leads to antigen-specific expansion of CD8+ T cells and antigen-specific, efficient tumor cell killing.

## Discussion

Vaccinations are successfully used in the therapy of cancer diseases leading to induction of (long-term) CD8+ T cell responses against the cancer cells [48, 49]. Most vaccines are administered intramuscularly or subcutaneously, however, mucosal administration is regarded to be more effective and has been successfully applied in several vaccination programs [50, 51]. This is due to the provocation of a mucosal immune response which is induced by local antigen processing and generates a sound cytotoxic T cell response, especially when administered via the upper respiratory tract [52]. The mucosa of the respiratory tract is well equipped with immune cells as it is a major potential entrance port for airborne pathogens and thus needs to be protected well. DCs are present at bronchial bifurcations and in the lung epithelium [53]. In the nasal mucosal tissue, DCs can be found concentrated in the nasal-associated lymphoid tissue and throughout the nasal epithelium [54]. In this context, nasal mucosal vaccination appears to be most attractive because it is a noninvasive, easy and painless applicable approach implying a greater patient compliance [24, 55, 56]. However, most antigens administered as mucosal vaccination failed to induce potent CD8+ T cell responses because of poor peptide penetration through the epithelial barrier or mucosal clearance [57]. It appears to be crucial that the antigen is specifically delivered by a particulate antigen carrier system [58, 59]. Thus, intranasal vaccination using appropriate antigen delivery systems such as CNPs have been shown to potently increase antigen uptake and induce T cell-mediated immunity [60–62]. Besides these promising achievements, parameters determining the immunogenicity have to be further elucidated in order to improve this strategy particularly for highly malignant diseases such as PDAC. Our study using CNPs has revealed that the size of CNPs (200–700 nm) does not considerably impact CNP uptake by distinct APCs, but is a critical determinant impacting APCs survival particularly that of DCs which are vital for T cell priming and activation. Thus, survival of DCs was highest after exposure to the smallest CNPs (90/10). While some studies also showed that APCs are able to take up and process particulate antigens between 20 nm and 3 μm without a clear preference for a specific size range [11], other studies clearly indicate that uptake of antigen-loaded CNPs by DCs and macrophages is dependent on particle size, concentration of the encapsulated antigen as well as exposure time of APCs towards the CNPs [63]. As outlined above, chitosan has been proven to be well suited for the delivery of nucleic acids and antigens in several studies [60–62]. Here, the advantage over other materials was attributed to its cationic nature and thus to the potential of CNPs to promote endosomal disrupture and release of their cargo into the cytosol [64]. However, the mechanism of uptake into immunocompetent cells such as DCs is not fully elucidated but is believed to be mediated by phagocytosis or macropinocytosis [65]. Own data indicated that upon uptake, CNPs remain in vacuole-like structures close to the cell membrane, which are not acidified [66].

For mucosal vaccination, a size above 100 nm seems to be preferred as smaller particles are quickly drained to the lymph nodes without interaction with the local immune system [67]. However, own research showed that there might be a size optimum around 200–400 nm depending on the target cell and the material the particle is composed of [20, 68]. Larger particles have the advantage that they also deliver a larger amount of cargo to the cell if they are taken up. However, cells also need to cope with a large amount of material which may hamper their viability. Unlike other polymer nanoparticles such as Poly(lactic-co-glycolytic-acid (PLGA) particles, which are slowly biodegradable and easy to disperse in liquid, CNPs consist of a hydrogel-like biopolymer. In aqueous environments, these nanoparticles exhibit a swollen, gel-like structure which results in sticky particle agglomerates and allows intense interaction with cellular surfaces as seen in particle uptake studies [66]. Imaging cytometry but not conventional flow cytometry analysis revealed that CNPs intensely stick to cell surfaces and cannot be washed away easily. CNPs can be prepared in an aqueous environment without the need of any organic solvent. This is beneficial especially with respect to biocompatibility. Furthermore, we demonstrated that exposure to 90/10-CNPs loaded with SIINFEKL peptide but not empty CNPs promotes a pro-inflammatory phenotype of DCs as indicated by elevated expression of pro-inflammatory cytokines such as TNF-α, IL-1β and IL-6. While in our system CNPs alone hardly impact the phenotype of already differentiated APCs, Oliveira et al. demonstrated that exposure of monocytes to chitosan during differentiation culture promotes polarization of a pro-inflammatory phenotype in macrophages and DCs [69]. These diverging results might be explained by the following facts: i) chitosan impacts rather polarization than effector function of already differentiated APCs. Hence, when APCs are already polarized to a certain phenotype chitosan alone is not potent enough to shift the polarization while when it is available as main polarization factor it promotes differentiation into a pro-inflammatory phenotype. ii) The pro-inflammatory effect of chitosan only manifests when the cells have been exposed to the polymer for a certain time. Oliveira et al. analyzed the impact of chitosan on monocyte polarization during a time period of 3–10 days with monocytes exhibiting a pro-inflammatory phenotype earliest after having cultured on chitosan for 7 days [69]. In contrast, we analyzed the phenotypic alterations in DCs having been exposed to chitosan for 5–48 hours.

Irrespective of whether chitosan is applied alone or in combination with peptides, promotion of a pro-inflammatory phenotype in APCs appears rather beneficial in the treatment of cancer diseases in order to overcome the immunosuppressive conditions in the patients and within the tumor. Despite the fact that not only APCs but also epithelial cells are able to take up 90/10-CNPs—albeit to a lesser extent as in coculture ~65% of DCs and only ~38% of H411 cells revealed uptake of FITC-conjugated CNPs–our data and other studies support the suitability of CNPs as vehicles of efficient uptake by DCs [70, 71]. Finally, our study revealed maximum SIINFEKL-MHC-I complex presentation at the cell surface of DCs after 5 hours. It has been already demonstrated by other groups, that cross-presentation of exogenous OVA antigens by DCs occurs rapidly, which was determined e.g. between 6 and 16 hours [72–74]. The strong decline in H-2Kb-bound SIINFEKL presentation in DC2.4 cells after 24 hours might be explained by the lack of further maturation stimuli (e.g. like LPS, TNF-α or Poly I:C) during the time of CNP stimulation. As shown by Kukutsch et al., the expression of antigen/H-2Kb complexes at the cell surface of DCs can be extended up to 72 hours when the cells are pretreated with LPS or Poly I:C [74].

Importantly, the particulate application of antigens is far more effective in the induction of an immune response than vaccination with soluble antigens as drug carrier systems may further efficiently encapsulate and protect sensitive antigens and preferably target the cargo to APCs [39, 54, 66, 67].

In conclusion, our study strongly supports the suitability of CNPs as antigen vehicle to elicit potent antigen-specific T cell responses being in line with other studies [63]. Our study clearly suggests CNPs particularly of small size as most potent antigen vehicle because we could demonstrate that i) CNPs are efficiently taken up by DCs, those APCs that are necessary for priming and activation of CD8+ T cells, ii) CNP delivered antigen is presented in the context of MHC-I by DCs, iii) DCs stimulated with CNP-loaded antigen lead to activation and expansion of tumor antigen-specific CD8+ T cells and finally to induction of a potent anti-tumor response. Future studies have to verify the therapeutic potential of CNPs as antigen delivery system in preclinical PDAC mouse models.

## Supporting information

S1 Fig Characterization of M1- and M2-macrophages. Polarization of human monocytes in M1- and M2-macrophages (Mᶲ) was confirmed by **A)** flow cytometric analysis of cell surface levels of CD68, CD14, CD16 (being similarly expressed on both cell populations), HLA-DR (being more expressed on M1-Mᶲ) and CD163 (being more expressed on M2-Mᶲ). Representative histograms from one out of five independent experiments are shown. In addition, **B)** relative mRNA levels of TNF-α, IL-8, IL-1β, IL-6 (being higher expressed in M1-Mᶲ) and TGF-β1 and IL-10 (being higher expressed in M2-Mᶲ) were determined by RT-qPCR. Expression levels were normalized to expression of the housekeeping gene GAPDH and normalized to values determined for M2-Mᶲ. Data are presented as mean + SEM of 3 independent experiments. *p<0.05.
(TIF)

S2 Fig CNPs do hardly alter expression of typical cell surface markers of human DCs. Human DCs were either left untreated or incubated with 100 μg/ml empty (90/10) or SIINFEKL-loaded 90/10-CNPs for 5, 24 and 48 hours. Then, CD80, CD86, HLA-DR and PD-L1 cell surface levels were determined by flow cytometry. Representative histograms from one out of three independent experiments are shown.
(TIF)

S1 Table Surface markers used for characterization and identification of cell populations by flow cytometry, imaging cytometry and immunofluorescence analyses. (DOCX)

## Abbreviations

APC: antigen presenting cells
CLSM: Confocal laser scanning microscopy
CMC: Carmellose-sodium
CNPs: chitosan nanoparticles
DC: dendritic cells
DDA: degree of deacetylation
GAPDH: Glycerinaldehyd-3-phosphat-Dehydrogenase
GM-SCF: Granulocyte-macrophage colony-stimulating factor
IL: Interleukin
M-CSF: Macrophage colony-stimulating factor
PDAC: pancreatic ductal adenocarcinoma
PDI: polydispersity index
PLGA: Poly(lactic-co-glycolytic-acid
TGF-β1: Transforming Growth Factor-beta 1
TNF-α: Tumor Necrosis Factor-alpha
