# Supplementary figures and images for: Chitosan nanoparticles as antigen vehicles to induce effective tumor specific T cell responses
Source: PLoS One. 2020 Sep 30;15(9):e0239369. doi: 10.1371/journal.pone.0239369 (PMC7526875; doi:10.1371/journal.pone.0239369)

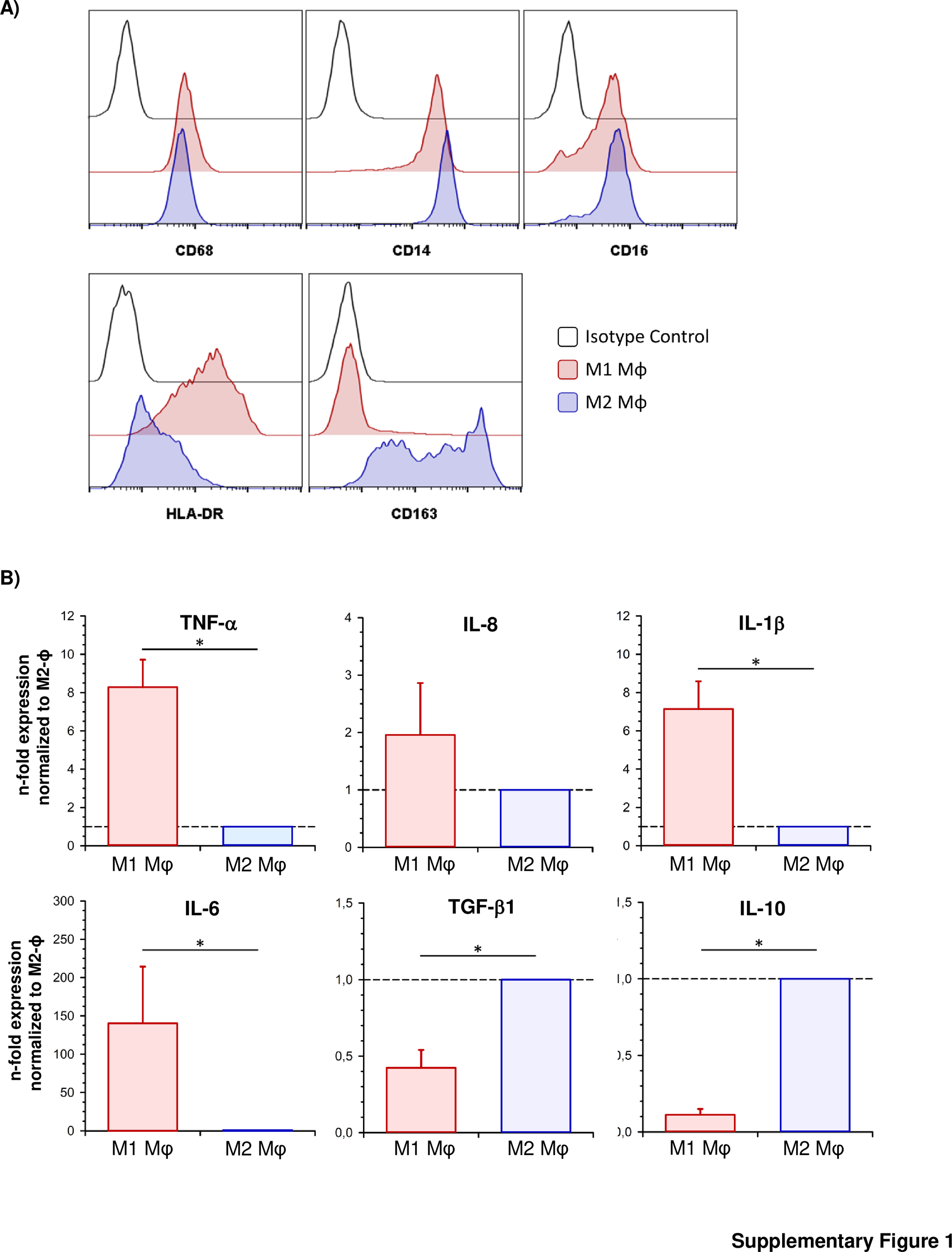

Supplement: S1 Fig — Polarization of human monocytes in M1- and M2-macrophages (Mᶲ) was confirmed by A) flow cytometric analysis of cell surface levels of CD68, CD14, CD16 (being similarly expressed on both cell populations), HLA-DR (being more expressed on M1-Mᶲ) and CD163 (being more expressed on M2-Mᶲ). Representative histograms from one out of five independent experiments are shown. In addition, B) relative mRNA levels of TNF-α, IL-8, IL-1β, IL-6 (being higher expressed in M1-Mᶲ) and TGF-β1 and IL-10 (being higher expressed in M2-Mᶲ) were determined by RT-qPCR. Expression levels were normalized to expression of the housekeeping gene GAPDH and normalized to values determined for M2-Mᶲ. Data are presented as mean + SEM of 3 independent experiments. *p<0.05. (TIF) [file pone.0239369.s001.tif]

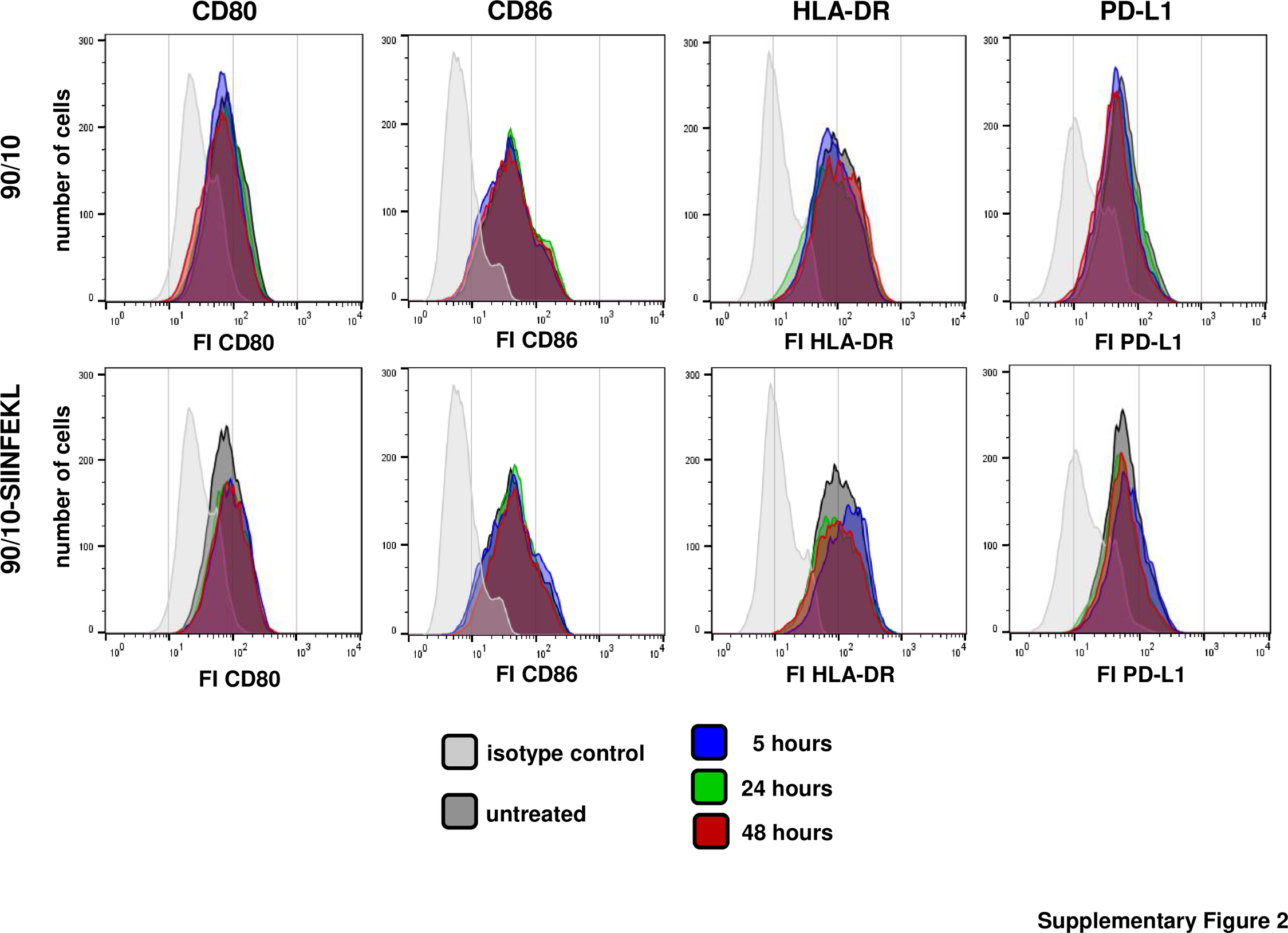

Supplement: S2 Fig — Human DCs were either left untreated or incubated with 100 μg/ml empty (90/10) or SIINFEKL-loaded 90/10-CNPs for 5, 24 and 48 hours. Then, CD80, CD86, HLA-DR and PD-L1 cell surface levels were determined by flow cytometry. Representative histograms from one out of three independent experiments are shown. (TIF) [file pone.0239369.s002.tif]
