# Supplementary material for: Chitosan nanoparticles as antigen vehicles to induce effective tumor specific T cell responses
Source: PLoS One. 2020 Sep 30;15(9):e0239369. doi: 10.1371/journal.pone.0239369 (PMC7526875; doi:10.1371/journal.pone.0239369)
Supplement: S1 Table — (DOCX) [file pone.0239369.s003.docx]

**S1 Table: Surface markers used for characterization and identification of cell populations by flow cytometry, imaging cytometry and immunofluorescence analyses**

| **Marker** | **Method** | | **Cell Population** |
| --- | --- | --- | --- |
| CD11c | | IF, IC | dendritic cells*, DC2.4 cells |
| CD14 | | FC | monocytes*, macrophages* |
| CD16 | | FC | monocytes*, macrophages* |
| CD163 | | FC | M2-polarized macrophages* |
| CD25 | | FC | activated OT-1 T cells |
| CD274 (PD-L1) | | FC | dendritic cells* |
| CD324 (E-Cadherin) | | IC | H441 cells |
| CD44 | | FC | activated OT-1 T cells |
| CD68 | | FC | monocytes*, macrophages* |
| CD69 | | FC | activated OT-1 T cells |
| CD80 | | FC | dendritic cells* |
| CD86 | | FC | dendritic cells* |
| CD8a | | FC | OT-1 T cells |
| HLA-DR | | FC | dendritic cells*, monocytes*, macrophages* |
| SIINFEKL H-2kb | | FC | DC2.4 cells |

FC = flow cytometry, IC = imaging cytometry, IF = immunofluorescence, * = primary human cells
